# Targeted gene panel for genetic testing of south Indian children with steroid resistant nephrotic syndrome

**DOI:** 10.1186/s12881-018-0714-6

**Published:** 2018-11-20

**Authors:** Annes Siji, K. N. Karthik, Varsha Chhotusing Pardeshi, P. S. Hari, Anil Vasudevan

**Affiliations:** 1 Division of Molecular Medicine, St. John’s Research Institute, Bangalore, India; 2 Department of Pediatric Nephrology, St. John’s Medical College Hospital, Bangalore, India

**Keywords:** SRNS, NGS, Targeted re-sequencing, Indian population

## Abstract

**Background:**

Steroid resistant nephrotic syndrome (SRNS) is a genetically heterogeneous disease with significant phenotypic variability. More than 53 podocyte-expressed genes are implicated in SRNS which complicates the routine use of genetic screening in the clinic. Next generation sequencing technology (NGS) allows rapid screening of multiple genes in large number of patients in a cost-effective manner.

**Methods:**

We developed a targeted panel of 17 genes to determine relative frequency of mutations in south Indian ethnicity and feasibility of using the assay in a clinical setting. Twenty-five children with SRNS and 3 healthy individuals were screened.

**Results:**

In this study, novel variants including 1 pathogenic variant (2 patients) and 3 likely pathogenic variants (3 patients) were identified. In addition, 2 novel variants of unknown significance (VUS) in 2 patients (8% of total patients) were also identified.

**Conclusions:**

The results show that genetic screening in SRNS using NGS is feasible in a clinical setting. However the panel needs to be screened in a larger cohort of children with SRNS in order to assess the utility of the customised targeted panel in Indian children with SRNS. Determining the prevalence of variants in Indian population and improvising the bioinformatics-based filtering strategy for a more accurate differentiation of pathogenic variants from those that are benign among the VUS will help in improving medical and genetic counselling in SRNS.

**Electronic supplementary material:**

The online version of this article (10.1186/s12881-018-0714-6) contains supplementary material, which is available to authorized users.

## Background

Steroid resistant nephrotic syndrome (SRNS) remains one of the most common intractable causes of end-stage renal disease (ESRD) in children with 50–70% of these children developing end-stage renal disease within 5–10 years of diagnosis [1]. The therapeutic options in SRNS are often inefficient, and complicated by significant toxicity adding to the associated morbidities, mortality and cost. There is now compelling evidence that children with pathogenic variations in the genes responsible for maintenance of podocyte structure and function form a distinct subgroup of Nephrotic Syndrome (NS) and these children are generally unresponsive to immunosuppression, but do not have post-transplantation recurrence [2, 3].

More than 53 single gene mutations specific to podocyte or associated with glomerular filtration barrier have been found to be associated with SRNS [4, 5]. Large multi centric studies including population of multiple ethnicities showed genetic mutations in about ~ 30% of SRNS patients with a higher proportion in infants and young children. Most mutations were observed in *NPHS2, WT1* and *NPHS1* genes [4, 6].

However, reports from India including from our center showed that the prevalence of *NPHS2* mutations is much lower in Indian population when compared with Europe and North American population [4% vs. 10.5–28%)] [7–12]. Kumar et al., reported low prevalence of *WT1* mutation in south Indian population, whereas we did not detect any mutation in *WT1* gene in 100 SRNS children [13, 14]. These data suggest that a traditional genetic testing using an algorithmic approach based on age of onset of NS to prioritize the genes to be sequenced by Sanger may not be useful [15, 16]. The above data also indicates the need for additional screening of genes implicated in SRNS in order to understand the genetic spectrum of SRNS in Indian population. Given the genetic heterogeneity and phenotypic variability in SRNS, Sanger sequencing is not a feasible approach for routine testing. Next-generation sequencing (NGS) technology is emerging as a cost-effective strategy to screen multiple genes in genetically heterogeneous diseases like SRNS [17].

The aim of our study was to check the feasibility of genetic diagnosis using targeted next-generation sequencing (NGS) approach in Indian children with SRNS. We report the initial results along with the challenges faced in the analysis and interpretation of sequencing data obtained by simultaneously sequencing 17 genes in 25 children with SRNS and 3 healthy individuals.

## Methods

### Subjects

The Institutional Ethics Committee approved the study and all participants were recruited after informed consent. Twenty five children with idiopathic SRNS (18 males: 7 females) as defined by standard guidelines were included [18]. Socio demographic information, clinical and treatment details were recorded in case record forms. All these children were previously analyzed by Sanger sequencing for all the exons of *NPHS2* and exon 8 and 9 of *WT1* genes [7, 14].We also included three subjects with pathogenic mutations in *NPHS2* reported previously to determine the sensitivity of the targeted-NGS method [7]. Three healthy individuals were included to check sequencing efficiency.

### Methods

Blood samples (5 ml) were collected from recruited patients and genomic DNA was extracted from peripheral blood leukocytes by the phenol chloroform method [19]. Quantity of the extracted DNA was estimated using Qubit fluorometric assay (Thermofisher scientific, MA, USA).

### Next-generation sequencing

For targeted next-generation sequencing, we selected a panel of 17 genes associated with SRNS based on their prevalence in clinically diagnosed SRNS patients and mutation frequency in the NS cohorts (Table 1) [4, 5, 20]. The genes selected for the panel accounted for 95–100% of the mutations in two large cohorts of SRNS one of which included Indian children [6, 21]. A total of 359 primers targeting the exonic regions of the selected 17 genes (307 exons) associated with nephrotic syndrome were designed using Ion Ampliseq Designer (Life Technologies, CA, USA). The amplicon size was designed in a range from 125 to 375 bp. The panel consisted of three primer pools amplicon size ranging from 125 to 375 bp and covering 99.6% exon of the selected genes. The uncovered region was mainly repeat rich region making primer designing difficult. An Ion Torrent adapter-ligated library was prepared using the Ion AmpliSeq Library Kit 2.0 (Life Technologies, CA, USA) by following the manufacturer’s protocol. Briefly, 10 ng of DNA was amplified by PCR using the premixed primer pool and Ion AmpliSeq HiFi master mix. After PCR, the amplified targets were treated with FuPa reagent to partially digest primer sequences and phosphorylate the amplicons. For adaptor ligation, amplicons from each sample were combined with a barcode adapter mix that contained Ion P1 adaptor and a unique Ion Xpress Barcode (Life Technologies, CA, USA). The unamplified libraries were purified using AMPure beads (Beckman Coulter, CA, USA) and the purified beads were amplified using Platinum PCR SuperMix High Fidelity and Library Amplification Primer Mix (Life Technologies, CA, USA). The amplified library was purified using AMPure beads. Library quantity and quality was determined using Qubit fluorometric assay and Agilent BioAnalyzer High-Sensitivity DNA kit (Agilent Technologies, CA, USA), respectively.

**Table 1:** Genes included in the targeted NS panel to screen genetic variant in Indian SRNS cohort (to be placed after Page 5)

Gene	Accession #	Disease	Inheritance	# exons covered	# exons not covered	# primer pairs
ACTN4^a^	NM_004924	Familial and sporadic SRNS (usually adult)	AD	21	–	25
ADCK4	NM_024876	SRNS	AR	13	1	15
CD2AP	NM_012120	FSGS/SRNS	AD/AR	18	–	20
COQ2	NM_015697	Mitochondrial disease/isolated nephropathy	AR	7	–	9
COQ6	NM_182476	NS + sensorineural deafness; DMS	AR	11	1	13
INF2	NM_022489	Familial and sporadic SRNS, FSGS-associated Charcot-Marie-Tooth neuropathy	AD	22	1	37
LAMB2	NM_002292	Pierson syndrome	AR	32	1	35
LMX1B	NM_002316	Nail patella syndrome; also FSGS without extrarenal involvement	AD	8	2	13
MYO1E	NM_004998	Familial SRNS	AR	28	–	28
NEIL1	NM_024608	childhood SRNS	AR	11	–	12
NPHS1	NM_004646	CNS/SRNS	AR	29	–	32
NPHS2^a^	NM_014625	CNS, SRNS	AR	8	–	10
PDSS2	NM_020381	Leigh syndrome	AR	8	–	9
PLCe1^a^	NM_016341	CNS/SRNS	AR	32	–	42
PTPRO	NM_030667	NS	AR	25	2	28
TRPC6	NM_004621	Familial and sporadic SRNS (mainly adult)	AD	13		18
WT1	NM_024426	Sporadic SRNS (children: may be associated with abnormal genitalia); Denys-Drash and Frasier syndrome	AD	10	–	13

*AD* autosomal dominant, *AR* autosomal recessive, *DMS* diffuse mesangial sclerosis, *ESRD* end-stage renal disease, *FSGS* focal segmental glomerulosclerosis, *NS* nephrotic syndrome, *SDNS* steroid-dependent nephrotic syndrome, *SRNS* steroid resistant nephrotic syndrome. ^a^Genes with a likely or known mutation, or a risk allele, in this cohort

Template preparation, emulsion PCR, and Ion Sphere Particles (ISP) enrichment were done using the Ion PGM Template OT2 400 kit (Life Technologies, CA, USA) according to the manufacturer’s instructions. Next-generation sequencing was carried out on Ion Torrent Personal Genome Machine sequencer (Life Technologies, CA, USA) using the Ion 318 and 314 Chips (Life Technologies, CA, USA) and Ion PGM Hi-Q Sequencing Kit (Life Technologies, CA, USA) according to the manufacturer’s instructions.

### Variant calling and annotation

The data from the both sequencing runs were analyzed using the Torrent Suite V5 analysis pipeline. Sequence reads were separated according to their barcodes. Human genome sequence (build GRCh37/hg19) was used as a reference sequence. For each individual barcode, the sequence reads were aligned to this reference sequence with a Torrent Mapping Alignment Program optimized to Ion torrent data using the default alignment algorithm and parameters. After alignment, the variants were annotated to determine their clinical significance by using a combination of frequency, structural prediction, or evidence-based data. The DNA variant regions were piled up with Torrent Variant Caller (TVC) plug-in software to identify missense, nonsense, frameshift, obligatory splice variants and short insertion/deletion (indels) across the targeted subset of the reference using germ-line parameters and low stringency settings. The output variant call format (VCF) file was then annotated using Ion Reporter Software v5.0 (Life Technologies, CA, USA) and variants were further investigated. All the variants were filtered based on their coverage (coverage> 30), variant effect (non-synonymous, frameshift, nonsense), location (to detect splice site variants) and allele frequency in public databases (ExAc (http://exac.broadinstitute.org/), and 5000 Exome (http://evs.gs.washington.edu/EVS/) < 1%). The filtered variants were visually examined using Integrative Genomics Viewer (IGV) software (http//www.broadinstitute.org/igv), to further filter out variants with possible strand-bias and variants within homopolymeric region. In silico analysis using Sorting Tolerant From Intolerant (SIFT) and Polymorphism Phenotyping v2 (Polyphen-2) tools was performed to predict the potential deleterious effect of the identified missense variants on protein function [22, 23]. Bioinformatics analysis of the strength of predicted splice site variants was performed with neural networks (NNSPLICE 0.9) [24]. The variants were classified as pathogenic, likely pathogenic, uncertain significance, likely benign, or benign according to the stringent criteria of American College of Medical Genetics and Genomics (ACMG) Standards and Guidelines and Sherloc rules [25, 26]. A scoring system developed by Karbassi et al. was used to determine the pathogenicity of VUS identified in this study [27].

The pathogenic and likely pathogenic variants were validated by Sanger sequencing using variant specific primers in patients as well as in healthy individuals (*n* = 30) (Additional file 1: Table S1).

## Results

### Demographic and clinical profile

The clinical details of 25 SRNS patients are presented in Table 2 with detailed phenotyping in Additional file 1: Table S2. The median age of onset of NS was 2.5 years- (0.58–16 years) with a median follow up of 2.5 years.. Majority of the patients were non-responsive to non steroidal immunosuppressant, with only 8 children demonstrating partial response to calcineurin inhibitors (Additional file 1: Table S2).

**Table 2:** Clinical characteristics of the South Indian nephrotic syndrome cohort

Characteristics		Total (*n* = 25) (%)
Sex	Male	18 (72)
	Female	7 (28)
Age at diagnosis	Median (years)	2.5 years
	Infantile (4–12 months)	3 (12)
	Early childhood (13 months −5 years)	16 (64)
	Late childhood (6–12 years)	4 (16)
	Adolescent (13–18 years)	2 (8)
Family history	Yes	7 (28)
	No	18 (72)
Parental consanguinity	Yes	5 (20)
	No	20 (80)
Steroid resistance	primary steroid resistance	24 (96)
	Secondary steroid resistance	1 (4)
Histopathology subtype	Focal segmental glomerulosclerosis (FSGS)	14 (56)
	Minimal change disease (MCD)	3 (12)
	Mesangial hypercellularity (MHC)	6(24)
	Diffuse mesangial sclerosis (DMS)	1 (4)
	Unknown	1 (4)
Renal outcome	Remission	2 (8)
	Persistent relapse	9 (36)
	Chronic Kidney disease Stage II-IV	4 (16)
	End stage renal disease	5 (20)
	Underwent renal transplant	1 (4)
	Dead	4 (16)

### Sequencing results

Two sequencing runs, containing 25 samples (23 patients and 2 healthy individual sample; 318 chip) and 4 samples (2 patients, 1 healthy individual sample and one human standard CEPH DNA sample; 314 chip) were performed. Total 854 M (Q20) and 172 M of Q20 data were obtained per 318 and 314 chips respectively and the coverage was comparable between runs. After filtering out polyclonal, low quality reads, and primer-dimers, the percentage of usable reads were 4.57 M and 0.788 M per 318 and 314 chips respectively (Additional file 1: Table S3). Combining the data derived from two runs, sequencing of the 17 glomerular disease gene panel generated a mean of 0.18 M reads per individual with mean read length of 214 bp. Only 10% of called bases had a quality score of <Q20; About 99% of these reads were mapped to the reference genome (hg19) and 93.9% of mapped reads were on target genes (Additional file 1: Table S4). A mean coverage of 442× was achieved for the genes across all individuals, with 93.1, 63.2 and 17% of the targets having minimum read depth of 20×, 100× and 500× respectively.

**Fig. 1:**
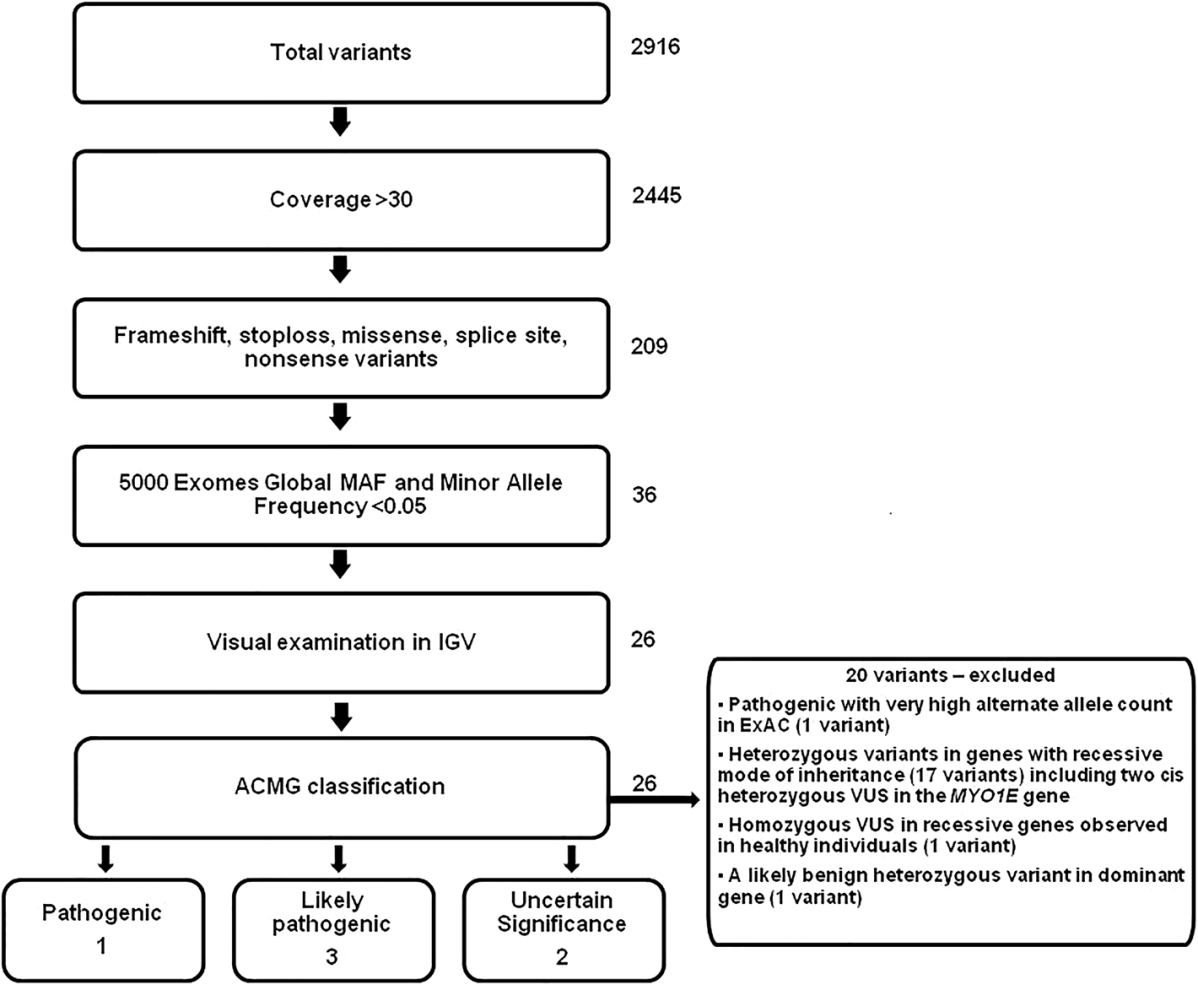
Flow chart of next generation sequencing variant filtration and annotation. The variants were filtered based on their coverage (minimum coverage of 20×), variant effect, dbSNP, ExAC, 500 exomes and 1000 Genome Project databases status. The filtered variants were visually examined using Integrative Genomics Viewer (IGV) software (http//www.broadinstitute.org/igv), to further filter out variants with possible strand-bias and variants that fall into homopolymeric region. All the filtered variants were annotated as per the ACMG guidelines

Overall, 2916 single-nucleotide variants (SNVs) and indels were identified in the 25 patients and 3 healthy individuals by Torrent Suite software V5, using default germline parameters. These variants were annotated and filtered using the Ion Reporter Software 4.4 with following parameters: inclusion of frameshift, stop loss, missense, nonsense variants and variants located in splice site with a minimum coverage of 20×. After the filtration, a total of 26 variants (23 missense, 2 nonsense and 1 splice site) were identified in 13 genes in 16 subjects (Fig. 1). Among these variants, 1 pathogenic *NPSH2* (R71X), 3 likely pathogenic [*PLCe1* (R752X), *NPHS1* (G968 V) and *NPHS*2 (splice site variant, g .179521737C > T)] and 2 VUS (*LMX1B* (V145 M) and *NPHS2* (H141Y) were considered clinically relevant. The remaining 20 variants not considered further for annotation included 15 heterozygous VUS in genes with recessive inheritance, two VUS (P973T and P995L) in *MYO1E* gene in a single patient (SRNS 60) in cis and a likely benign variant (R877Q) in *INF2* gene. A homozygous VUS in *PLCe1* (G222R) gene in SRNS was also excluded from further annotation, as it was observed in a healthy individual. A variant in *ACTN4* gene (R310Q) was excluded from clinically relevant list although it was classified as likely pathogenic based on ACMG criteria. This variant has a very low allele frequency in ExAC database and also has been reported in probands of families with FSGS and individuals with sporadic FSGS [0.0074 (8/1084) controls 0.016 (3/192) sporadic FSGS] [28]. Besides, podocyte transient transfection assay indicates that the mutation inhibited the complex formation between α-actinin-4 and CLP36 causing the podocyte defect although the precise pathways involved were not identified [29]. However, a large number of alternate alleles (*n* = 1426) have been identified at the same position in general population Although global allele frequency of p.R310Q variant in *ACTN4* was < 1%, total allele count was higher (3138) in gnomAD database (Updated version of ExAC, http://gnomad.broadinstitute.org/variant/19-39207742-G-A). As per the Sherloc rule (EV0161, https://www.ncbi.nlm.nih.gov/pubmed/28492532), variants with allele count > 8, is considered as high allele count and the variant is classified as benign. Therefore although the based on the ACMG criteria p.R310Q variant in ACTN4 was classified as pathogenic, it was considered as benign based on the improved and robust variant classification guidelines of Sherloc.

The pathogenic variant in *NPSH2* (R71X) gene was observed in a pair of sibling (8%). The likely pathogenic variants in *PLCe1* (R752X), *NPHS1* (G968 V) and *NPHS*2 (splice site variant, g .179521737C > T) genes were identified in one subject each (4%) (Table 3). All these variants were novel except for the *NPHS*2 variant (R71X) [30]. Of the 2 variants, identified by Sangers sequencing previously, one variant (H141Y) was not present in the final filtered variants. A review of the NGS data indicated that the variant was identified by the sequencing but was filtered because of the stringent variant filtration settings (minimum read depth of 30×). A total of 18 reads was obtained for this variant H141Y in *NPHS2*. The pathogenic and likely pathogenic variants were validated using Sanger sequencing in the respective patients and 30 healthy individual samples and no false positives were identified. The pathogenicity score of two variants (*LMX1B*; heterozygous, V145 M, and*NPHS2*; homozygous, H141Y) classified as variants of uncertain significance (VUS) indicated that they could be pathogenic in nature and needed to be explored further for their causality in SRNS (Additional file 1: Table S5).

**Table 3:** Description of pathogenic and likely pathogenic variants identified in the south Indian steroid resistant nephrotic syndrome cohort

Patient ID	Gene	Zygosity	Nucleotide Change	Amino acid Change	Mutation type	ACMG classification	Prediction							ExAC		
							dbSNP (Build 146)	SIFT	PolyPhen-2	fathmm	Mutation Taster	splicing Predictions-NNSPLICE,ASSP	Alternative allele count	Allele number	No.of h/hemi	Allele frequency
SRNS20, SRNS76	*NPHS2*	Homozygous	c.211C>T	p.R71X	Nonsense	Pathogenic	NA	NA	NA	NA	NA	NA	0	0	0	0
SRNS123	*PLCe1*	Homozygous	c.2254C>T	p.R752X	Nonsense	Likely Pathogenic	NA	NA	NA	NA	NA	NA	1	120380	0	0
SRNS83	*NPHS2*	Homozygous	g.179521737C>T		Splice site	Likely Pathogenic	NA	NA	NA	NA	NA	Y,Y	0	0	0	0
SRNS13	*NPHS1*	Homozygous	c.2903G>T	p.G968V	Missense	Likely Pathogenic	NA	NA	Y (Possibly damaging - 0.887)	Y	Disease causing	NA	0	0	0	0

### Genotype –phenotype correlation of disease-causing variants in the cohort

The clinical features and the renal histology were similar between those with pathogenic or likely pathogenic variants. Response to immunosuppressive treatments was not significantly different between those with pathogenic or likely pathogenic variants and those without an abnormal variant. The homozygous nonsense R71X variant in *NPHS*2 gene was identified in two siblings (patient SRNS20 and SRNS76). The siblings presented with NS at age of 3.5 and 2.5 years respectively and both showed FSGS on biopsy. Both subjects showed no response to calcineurin inhibitors. The elder sibling (patient SRNS20) progressed to ESRD by the age of 5 years and died at the age of 6 years with sepsis. The younger sibling currently aged 4.5 years (patient SRNS76) is in CKD stage 3 (Additional file 1: Table S2). Their parents were heterozygous for the point mutation (data not shown). A likely pathogenic homozygous nonsense variant R752X in *PLCe*1 gene was identified in SRNS123 in whom renal biopsy showed DMS (Additional file 1: Table S3). This child presented with symptoms of NS at the age of 1.5 years had renal dysfunction at the time of diagnosis and progressed to ESRD within a year of diagnosis. Similar histopathology has been reported with pathogenic variants in *PLCe1* gene [31]. A splice site likely pathogenic variant was observed in *NPHS2* gene in patient 83 who also had a homozygous VUS in the same gene (H141Y missense, both parents are heterozygous for this particular variant). This child manifested SRNS at the age of 1.2 years, showed FSGS on renal biopsy and progressed to ESRD, 2 years after diagnosis. SRNS 13 was identified to have the homozygous recessive variant (G968 V) in the *NPHS1* gene. The child was diagnosed as SRNS at age of 10 months, with the biopsy report revealing MHC and is in remission at last follow up. Patient 73 in whom a heterozygous variant in *LMX1B* (dominant inheritance) was observed manifested SRNS at the age of 3.5 years with FSGS on biopsy and progressed to ESRD within 7 years of diagnosis. The risk score suggests pathogenicity.

## Discussion

Identifying the cause of SRNS is of great importance as it helps in preventing unnecessary exposure to immunosuppressants and their adverse effects, besides establishing a molecular diagnosis and clear prognosis. It also enables targeted treatment as in case of children with pathogenic variants identified in gene encoding enzymes of the co-enzyme Q 10 biosynthesis who are amenable to treatment with coenzyme Q 10 [32].

We report the results of sequencing for molecular diagnosis of SRNS in Indian children by screening 17 genes wherein pathogenic variant in *NPHS2* gene was identified in 8% patients. Siblings carrying this variant along with the patient 83 carrying the *NPHS2* variant H141Y were included as positive samples to check the sensitivity of the present assay. Both these variants were detected (although variant H141Y was initially missed due to low read depth) and no spurious pathogenic mutations were found in any of these samples indicating 85% sensitivity for the assay. Beside these known variants, 3 novel likely pathogenic variants were identified in 3 patients (12%) who were previously sequenced for *NPHS2* and *WT1* genes. These findings demonstrate the utility of NGS in a clinical setting since it allows for rapid and simultaneous screening of multiple SRNS associated genes instead of prioritizing specific genes for genetic testing.

The targeted gene panel was developed based on the results from two largest SRNS cohorts one of which included Indian children with SRNS. The targeted panel included 17 genes which explained the genetic basis in > 95% of children with SRNS in these two cohorts. Previous studies using the targeted multi-gene sequencing to analyze the exon and intron boundaries of genes associated with SRNS in various populations identified mutations in ~ 30% of the patients [4–6, 21, 33–37]. In the present study, disease causing variants were identified in 20% of the cohort which is lesser than that expected probably due to small number of patients included in the cohort.

The most common disease causing variants were identified in the *NPHS2*, *WT1*, and *NPHS1* genes in the Podonet cohort (1174 patients from 21 countries; included 9 Indian patients = 0.7%), in 1783 unrelated, multinational cohort and in the UK cohort [21]. This in contrast to the Chinese population, wherein the disease causing variants were also identified in *ADCK4* gene (6.67%), in addition to *NPHS1*, *WT1*, and *NPHS2* genes [37]. In the present study, although the cohort size was small, disease causing variants were identified in *NPHS2* (12%) *NPHS1* (4%) and *PLCe*1 (4%) genes indicating that the genes with variants causing SRNS varies significantly according to ethnic background. While this study and our previous study indicate that *NPHS2* gene is the most common mutated gene in Indian population [7], we also identified *NPHS1* and *PLCe1* genes mutations that would not have been considered in the conventional genetic testing algorithms for SRNS using Sanger sequencing.

All the pathogenic variants were identified in genes associated with recessive Mendelian inheritance, as most of the children (64%) in the cohort developed SRNS at an early age (< 5 years). The age of onset in our study correlated with risk for an as reported in other studies [6, 16]. Surprisingly, we did not find any pathogenic variants in infantile group. This is contrast to the findings from other studies where in ~ 66.3% of SRNS cases (onset between 0 and 1 year) is due to the mutation in one of following four genes: *NPHS1, NPHS2, LAMB2, or WT1* [38]. This indicates that additional SRNS associated genes needs to be screened in this group.

It is well known that SRNS exhibits significant inter and intra familial variability. The use of NGS allows to study the influence of disease causing variants in multiple genes on phenotype variability [33]. In the present cohort, two siblings with identical pathogenic variant (*NPHS2* R71X; SRNS20 and SRNS76) showed different clinical course. The variability in the clinical phenotype of patients carrying the same variant indicate an environmental factor or a possible second-site genetic modification, whereby pathogenic variants in a second gene might modulate the penetrance and/or expressivity of recessive mutations in a primary locus. Although in the siblings we did identify additional variant (R408Q) in *NPHS1*, it was heterozygous and classified as begnin by both ACMG and Karbassi et al. variant scoring system [25, 27]. In patient 83, two variants in the *NPHS2* gene (splice site, g.179521737C > T and missense H141Y) were identified. The splice site variant was classified as likely pathogenic while the H141Y variant was classified as VUS, with the risk score suggesting pathogenic nature. It is difficult to predict which variant is contributing to the disease development in this child. In order determine the role of multiple variants on the phenotypic variability we need to compare patients with different genotype combinations in the various cohorts that have been studied.

The main barrier to determine the pathogenicity of a variant is absence or limited functional testing of variants discovered to identify specific variants that results in dysfunction of the protein product. For example, a novel homozygous variant R752X, in *PLCe1* gene in patient 123 was classified as likely pathogenic instead of pathogenic. Based on the clinical findings and histopathology of patient 123, it is evident that *PLCe1* gene variant can potentially be attributed to the disease development in this patient. However, lack of data which would help with the segregation of alleles in cases and the reference population and absence of functional data, we were unable to classify this variant as pathogenic.

Secondly, guidelines to annotate the heterozygous variants in dominant genes are not very clear. For example the novel *LMX1B* gene variant V145 M with low allele frequency was predicted to be pathogenic in nature as per the Karbassi scoring algorithm but still classified as VUS as per the ACMG criteria. Further functional studies are required to confirm the effect of this variant on protein function and disease phenotype. Since little robust data is available upon which to base an assessment of causality in case of VUS, reporting, genetic and medical counseling can be complex and challenging. There is no consensus on optimal strategies to report such findings and for clinician to communicate them with parents. Counselling parents with an affected child with a VUS is even more challenging in a prenatal setting as quantifying the attributable risk of developing the disease is not possible if the variant is prospectively detected in the unborn fetus. Hence developing appropriate and effective clinical approaches to this challenge including additional training to clinicians in pretest counseling and consenting, interpretation of results and communication of results to the parents is essential. Besides, integrating the data from this study with large publically accessible phenotype and genotype data may help in ascertaining the role of novel variants in disease development and also determine the role of multiple variants on the phenotypic variability.

This study is unique as it is the first Indian study using well phenotyped SRNS cohort and NGS technology for the genetic diagnosis of SRNS. However it had few limitations such as non-random sample selection (majority of the patients were early childhood onset) and selection of small number of patients from a single center. As parental DNA was not available we could not perform segregation studies in the familial cases except in one family.

## Conclusions

In conclusion, we demonstrated the feasibility of genetic screening using a targeted gene panel in a clinical setting. However, a larger number of children with SRNS needs to be screened in order to know the genetic profile as well as determine the utility of customizing targeted gene panel to screen Indian children with SRNS. Such screening will help the clinician in better prognostication and rationalizing treatment of SRNS patients. However, there were challenges in the interpretation of variants and uncertainty of some results. Improving bioinformatics-based filtering strategy will help in differentiating pathogenic variants from those that are benign among VUS.

## Additional file

Additional file 1: **Table S1.** Details of primers used for Sanger sequencing to validate the variants identified by next generation sequencing. **Table S2.** Detailed Clinical profile of the South Indian steroid resistant nephrotic syndrome cohort. **Table S3.** Ion PGM next-Generation Sequencing run summary. **Table S4.** Summary of per sample NGS data output and quality in Indian steroid resistant Nephrotic syndrome cohort. **Table S5.** Pathogenicity risk score of the Variant of unknown significance (VUS) (XLSX 25 kb)

## Abbreviations

ACMG: American college of medical genetics
CEPH: Centre d’Etude du polymorphisme
CKD: Chronic kidney disease
DMS: Diffuse mesiangial sclerosis
DNA: Deoxyribo nucleic acid
ESRD: End stage renal disease
FSGS: Focal segmental glomerulosclerosis
IGV: Integrative genomics viewer
ISP: Ion sphere particles
MCD: Minimal change disease
MHC: Mesiangial hypercellularity
NGS: Next generation sequencing
NS: Nephrotic syndrome
SIFT: Sorting tolerant from intolerant
SNV: Single nucleotide variant
SRNS: Steroid resistant nephrotic syndrome
TVC: Torrent variant calling
VCF: Variant call format
VUS: Variants of unknown significance
